# Development and validation of a novel nomogram for predicting long-term survival in patients with decompensated cirrhosis undergoing TIPS

**DOI:** 10.3389/fmed.2026.1766352

**Published:** 2026-04-07

**Authors:** Zhimeng Jiang, Wencai Lai, Jianguo Chu, Zheyi Han, Baojie Wei, Zhibo Xia, Tongzhen Zhang, Nianjun Xiao, Shoubin Ning

**Affiliations:** 1 Graduate School of Hebei North University, Zhangjiakou, Hebei, China; 2 Department of Gastroenterology, Air Force Medical Center, Chinese People’s Liberation Army, Beijing, China

**Keywords:** decompensated cirrhosis, nomogram, risk stratification, survival prediction, TIPS

## Abstract

**Background and aims:**

Transjugular intrahepatic portosystemic shunt (TIPS) is a critical intervention for complications of decompensated cirrhosis. However, traditional scoring systems like MELD and CTP have limitations in predicting long-term post-TIPS survival, particularly in populations with viral hepatitis. This study aimed to develop and validate a novel nomogram to predict 1-, 3-, and 5-year overall survival (OS) in patients with decompensated cirrhosis undergoing TIPS.

**Methods:**

We conducted a single-center retrospective study enrolling 409 patients with decompensated cirrhosis who received their first TIPS treatment between January 2017 and December 2023. Patients were randomly assigned to a training cohort (*n* = 286) and a validation cohort (*n* = 123). Independent prognostic factors were identified using multivariate Cox proportional hazards regression to construct a nomogram. Model performance was evaluated using time-dependent receiver operating characteristic (ROC) curves, calibration plots, and decision curve analysis (DCA), and compared with MELD-Na and CTP scores.

**Results:**

Six independent predictors were identified: age, serum ammonia, total cholesterol, total bilirubin, albumin, and creatinine. The nomogram demonstrated superior discrimination, the areas under the ROC curve (AUCs) for 1-, 3-, and 5-year OS were 0.79, 0.82, and 0.84 in the training cohort, and 0.81, 0.75, and 0.80 in the validation cohort, respectively. Calibration curves showed excellent agreement between predicted and observed survival, and DCA indicated significant clinical net benefit.

**Conclusion:**

We developed a robust nomogram integrating hepatic, renal, and metabolic indicators to predict long-term survival in post-TIPS patients. This model offers superior accuracy compared to traditional scoring systems, facilitating better risk stratification and individualized clinical decision-making.

## Introduction

Viral hepatitis and other chronic liver diseases are the primary causes of liver cirrhosis and hepatocellular carcinoma, posing a severe public health challenge, particularly in East Asia (1). Decompensated cirrhosis is frequently accompanied by portal hypertension, which precipitates a series of fatal complications. Notably, the six-week mortality rate following the first episode of esophageal-gastric variceal bleeding (EGVB) can reach 15–20% (2). Furthermore, approximately 5–10% of patients with ascites develop refractory ascites within 1 year, associated with a one-year mortality risk approaching 50% (3). These complications are the principal factors contributing to poor prognosis and significantly elevated mortality risks in this patient population.

Transjugular intrahepatic portosystemic shunt (TIPS) is a minimally invasive interventional technique that establishes an artificial shunt between the hepatic and portal veins to effectively reduce portal pressure. It is currently a cornerstone modality for controlling and treating complications related to portal hypertension in cirrhosis (4). Extensive clinical practice has confirmed the efficacy of TIPS in preventing variceal rebleeding and treating refractory ascites. Particularly for high-risk patients with acute variceal bleeding, early TIPS placement has been shown to significantly improve survival rates (5).

However, despite the maturation of TIPS technology, significant disparities in long-term post-procedural survival persist. Some patients derive limited survival benefit or experience increased post-operative complications. Therefore, accurate pre-operative mortality risk assessment is critical for optimizing TIPS candidate selection and improving overall prognosis. Currently, the model for end-stage liver disease (MELD) and the Child–Turcotte–Pugh (CTP) score are widely used in clinical practice. While they reflect hepatic functional reserve and disease severity, their accuracy in predicting long-term survival after TIPS remains controversial (6, 7), as they may not comprehensively capture all complex factors influencing prognosis (8).

Given the limitations of existing scoring systems, the development of more precise, individualized prediction tools has become a research hotspot (9). In recent years, several researchers have attempted to construct specialized prognostic models for post-TIPS outcomes (10). However, most of these models are based on Western populations with mixed etiologies and lack specificity for the demographic characteristics of the Chinese population. Consequently, this study systematically reviewed pre-operative clinical data of patients with decompensated cirrhosis undergoing TIPS to identify independent risk factors for long-term survival. We aimed to construct and verify a nomogram prediction model based on readily available pre-operative indicators, with the expectation of providing a more reliable basis for risk stratification and individualized treatment decision-making in decompensated cirrhosis patients prior to TIPS.

## Methods

### Study design and patient selection

This was a single-center, retrospective cohort study conducted at the Air Force Medical Center, PLA. We consecutively enrolled patients diagnosed with decompensated cirrhosis who underwent their first TIPS procedure at our center between January 2017 and December 2023. The study protocol was approved by the Ethics Committee of the Air Force Medical Center, PLA [Approval No. Kongte (Scientific Research) No. 2025-38-PJ01].

Inclusion criteria were: (1) Patients with a definite diagnosis of decompensated cirrhosis based on clinical, imaging, or pathological evidence; (2) Patients undergoing primary TIPS treatment for EGVB or refractory ascites. Exclusion criteria were: (1) Patients with severe infection, malignancy, hematological disorders, severe cardiopulmonary insufficiency, or other non-cirrhotic conditions leading to poor prognosis; (2) History of liver transplantation; (3) Missing key clinical data.

### Data collection and definitions

Baseline clinical data were collected via the hospital’s electronic medical record system. The collection window was within 1 week prior to the TIPS procedure. (1) Demographic and clinical data: Age, gender, body mass index, smoking history, alcohol consumption history, and comorbidities (diabetes, hypertension). (2) Medical history: History of gastrointestinal bleeding, ascites, and hepatic encephalopathy (HE). (3) Laboratory indicators: Complete blood count (white blood cell count, red blood cell, hemoglobin, platelets), liver function tests (alanine aminotransferase, aspartate aminotransferase), total bilirubin (TBIL), albumin, total protein (TC), renal function (urea nitrogen, creatinine), electrolytes (serum sodium), lipid profile, coagulation function (prothrombin time, activated partial thromboplastin time), serum ammonia, and cholinesterase. (4) Clinical scores: MELD score = 11.2 × ln(international normalized ratio) + 9.57 × ln(creatinine, mg/dL) + 3.78 × ln(total bilirubin, mg/dL) + 6.43 (11), MELD-Na = MELD + 1.32 × (137-Na) − [0.033 × MELD × (137-Na)], CTP score: total bilirubin, albumin, prothrombin time, ascites, HE (12).

### Follow-up and study endpoints

The primary endpoint of this study was overall survival (OS), defined as the time from the date of TIPS procedure to the date of death from any cause or the last follow-up. All patients were routinely followed up via hospitalization records or telephone interviews until death or the study cutoff date (March 1, 2025).

### Statistical analysis

#### Baseline characteristics

Continuous variables were described as mean ± standard deviation or median interquartile range depending on distribution and compared using the independent sample *t*-test or Mann–Whitney *U* test. Categorical variables were expressed as frequencies and percentages [*n* (%)] and compared using the chi-square test or Fisher’s exact test.

#### Factor screening and model construction

The primary observation endpoints were 1-, 3-, and 5-year OS post-TIPS. In the training set, univariate Cox regression was first used to screen potential prognostic variables. Variables with *p* < 0.05 in the univariate analysis were entered into a multivariate Cox proportional hazards regression model. Stepwise regression was employed to identify independent risk factors affecting survival.

Based on the independent predictors identified in the multivariate analysis—age, serum ammonia, TC, TBIL, albumin, and creatinine—a nomogram was constructed to predict 1-, 3-, and 5-year survival probabilities. Multicollinearity was assessed using the variance inflation factor.

#### Model validation

Internal validation was performed. Time-dependent receiver operating characteristic (ROC) curves were plotted, and the area under the curve (AUC) for 1 year, 3 years, and 5 years was calculated to evaluate predictive performance at specific time points. AUC of the new model was compared with those of MELD-Na and CTP scores. Calibration curves for 1 year, 3 years, and 5 years were plotted to assess the consistency between predicted and observed survival probabilities.

Based on the total nomogram score, the median was used as the optimal cutoff value to stratify all patients into high-risk and low-risk groups. Kaplan–Meier survival curves were plotted, and the log-rank test was used to compare OS differences. Decision curve analysis (DCA) was employed to evaluate the clinical net benefit at different threshold probabilities. All statistical analyses were performed using R software (version 4.3.3). A two-sided *p*-value <0.05 was considered statistically significant.

## Results

### Patient baseline characteristics and cohort allocation

**Figure 1:**
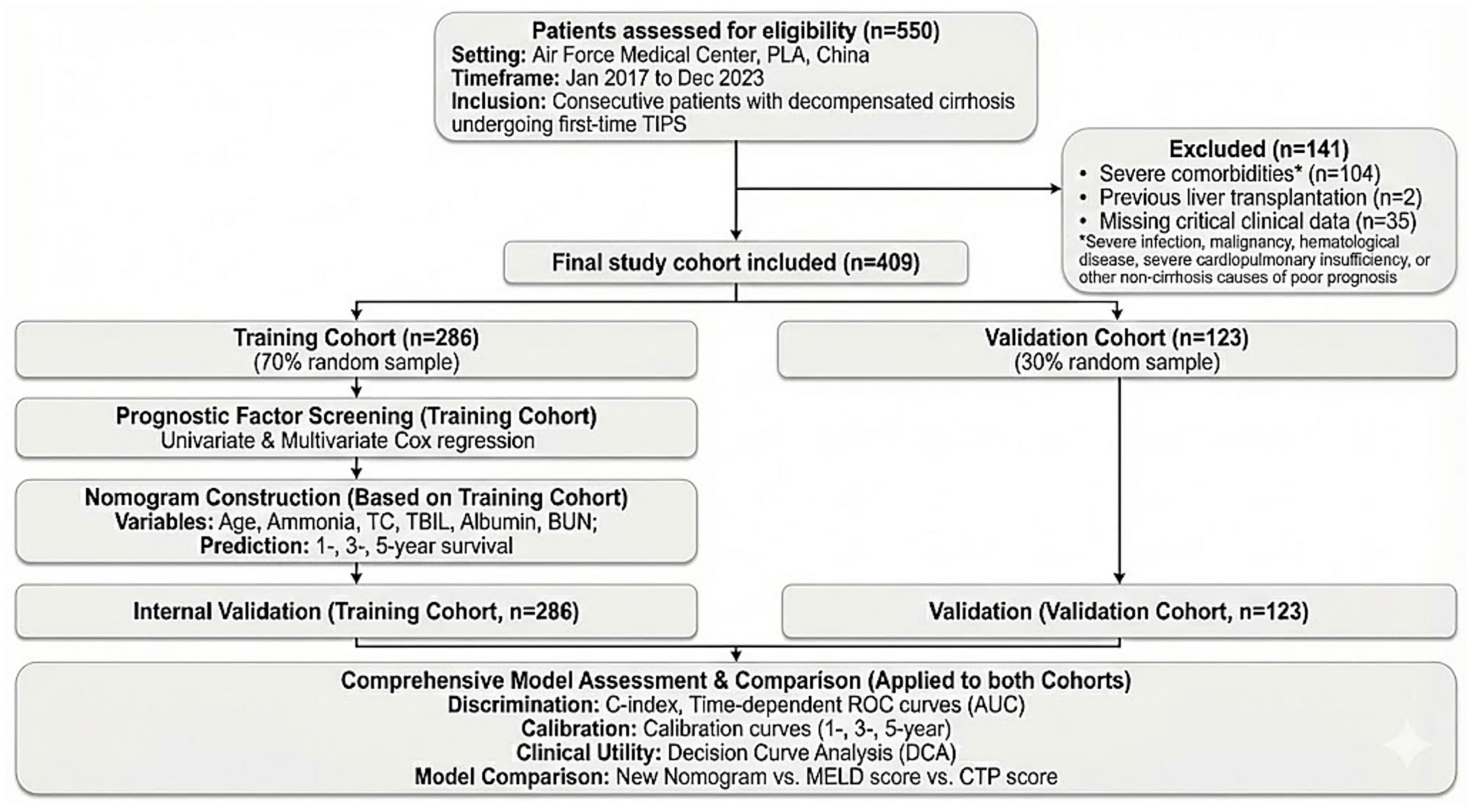
Flowchart of patient enrollment and study design.

Initially, 550 patients were evaluated. After excluding 141 patients based on the criteria, the final study cohort comprised 409 patients (Figure 1). Patients were randomly assigned (7:3 ratio) to a training cohort (*n* = 286) and a validation cohort (*n* = 123). As shown in Table 1, there were no statistically significant differences in baseline demographics, clinical characteristics, or laboratory indicators between the two cohorts (all *p* > 0.05), indicating a balanced distribution suitable for unbiased external validation.

**Table 1:** Baseline characteristics of the study population.

Characteristic	Total patients (*n* = 409)	Training set (*n* = 286)	Validation set (*n* = 123)	*p*-value
Age (years)	54.32 ± 12.84	54.04 ± 12.24	54.96 ± 14.16	0.508
Body mass index (kg/m^2^)	23.37 ± 13.17	23.82 ± 15.62	22.31 ± 2.94	0.288
Hemoglobin (g/L)	91.27 ± 26.64	90.49 ± 26.62	93.10 ± 26.69	0.363
Serum sodium (mmol/L)	138.44 ± 7.77	138.90 ± 3.81	137.37 ± 12.90	0.068
Total cholesterol (mmol/L)	3.77 ± 11.66	3.99 ± 13.91	3.26 ± 1.27	0.561
HDL-C (mmol/L)	0.90 ± 0.35	0.89 ± 0.34	0.90 ± 0.38	0.894
LDL-C (mmol/L)	1.77 ± 0.67	1.75 ± 0.62	1.80 ± 0.76	0.487
Albumin (g/L)	36.11 ± 5.54	35.99 ± 5.34	36.38 ± 6.00	0.522
MELD score	10.87 ± 4.50	10.80 ± 4.32	11.03 ± 4.91	0.634
MELD-Na score	10.56 ± 4.17	10.45 ± 4.22	10.97 ± 4.57	0.516
CTP score	6.90 ± 1.66	6.88 ± 1.59	6.94 ± 1.81	0.715
White blood cell count (×10^9^/L)	2.80 (1.95, 4.75)	2.88 (1.91, 4.75)	2.80 (2.00, 4.76)	0.807
Red blood cell (×10^9^/L)	3.19 (2.63, 3.75)	3.17 (2.62, 3.72)	3.21 (2.66, 3.78)	0.578
Platelet count (×10^9^/L)	72.00 (46.00, 109.00)	74.00 (48.00, 113.50)	66.00 (46.00, 95.00)	0.116
Neutrophil (×10^9^/L)	1.70 (1.12, 2.90)	1.79 (1.10, 2.97)	1.60 (1.14, 2.52)	0.528
Lymphocyte (×10^9^/L)	0.60 (0.40, 0.93)	0.61 (0.40, 1.00)	0.58 (0.40, 0.90)	0.285
Monocyte (×10^9^/L)	0.25 (0.16, 0.41)	0.26 (0.16, 0.44)	0.24 (0.16, 0.38)	0.401
Urea nitrogen (mmol/L)	5.40 (4.20, 7.50)	5.30 (4.20, 7.22)	5.50 (4.00, 7.85)	0.587
Creatinine (μmol/L)	62.00 (50.50, 78.00)	62.00 (50.12, 78.00)	61.00 (51.50, 77.55)	0.616
Triglyceride (mmol/L)	0.76 (0.60, 1.00)	0.73 (0.60, 0.99)	0.78 (0.61, 0.99)	0.361
Alanine aminotransferase (U/L)	18.90 (13.00, 27.00)	19.00 (13.00, 27.00)	18.00 (13.00, 26.00)	0.611
Aspartate aminotransferase (U/L)	27.00 (21.00, 38.00)	27.00 (21.40, 38.00)	26.00 (19.35, 37.40)	0.489
Total bilirubin (μmol/L)	20.30 (14.00, 31.20)	20.30 (13.72, 31.12)	20.30 (15.20, 31.25)	0.383
Total protein (g/L)	63.55 (57.99, 69.50)	63.30 (57.99, 69.20)	63.97 (57.90, 69.75)	0.308
Alkaline phosphatase (U/L)	79.00 (57.00, 111.00)	78.50 (57.00, 109.75)	80.00 (57.50, 117.00)	0.709
Gamma-glutamyl transferase (U/L)	30.00 (18.00, 61.30)	29.00 (18.77, 58.50)	36.00 (17.50, 66.50)	0.485
Total bile acid (μmol/L)	22.00 (10.00, 50.00)	22.90 (10.00, 50.22)	19.30 (10.45, 47.50)	0.694
Cholinesterase (U/L)	135.00 (93.00, 184.00)	133.00 (90.00, 180.00)	139.00 (100.00, 201.50)	0.366
Serum ammonia (μmol/L)	56.00 (40.00, 76.00)	56.00 (40.00, 75.00)	56.00 (42.00, 81.50)	0.311
lactate dehydrogenase (U/L)	188.00 (157.00, 224.00)	190.00 (159.00, 223.00)	184.50 (153.50, 224.50)	0.290
Prothrombin time (ser)	14.00 (12.70, 15.90)	14.10 (12.83, 16.08)	13.70 (12.50, 15.20)	0.106
APTT (ser)	33.80 (30.90, 36.70)	33.65 (30.72, 36.50)	34.00 (31.80, 37.05)	0.230
Gender, *n* (%)				0.806
Male	263 (64.30)	185 (64.69)	78 (63.41)	
Female	146 (35.70)	101 (35.31)	45 (36.59)	
Smoking, *n* (%)				0.704
No	294 (71.88)	204 (71.33)	90 (73.17)	
Yes	115 (28.12)	82 (28.67)	33 (26.83)	
Drink, *n* (%)				0.070
No	287 (70.17)	193 (67.48)	94 (76.42)	
Yes	122 (29.83)	93 (32.52)	29 (23.58)	
Diabetes, *n* (%)				0.655
No	327 (79.95)	227 (79.37)	100 (81.30)	
Yes	82 (20.05)	59 (20.63)	23 (18.70)	
Etiology, *n* (%)				0.593
Viral hepatica	194 (47.43)	140 (48.95)	54 (43.90)	
Alcoholic hepatitis	57 (13.94)	42 (14.69)	15 (12.20)	
Autoimmune hepatitis	23 (5.62)	14 (4.90)	9 (7.32)	
Portal vein cavernous degeneration	135 (33.01)	90 (31.47)	45 (36.59)	
Hypertension, *n* (%)				0.966
No	332 (81.17)	232 (81.12)	100 (81.30)	
Yes	77 (18.83)	54 (18.88)	23 (18.70)	
Previous history of gastrointestinal hemorrhage, *n* (%)				0.945
No	104 (25.43)	73 (25.52)	31 (25.20)	
Yes	305 (74.57)	213 (74.48)	92 (74.80)	
Previous history of ascites, *n* (%)				0.593
No	302 (73.84)	209 (73.08)	93 (75.61)	
Yes	107 (26.16)	77 (26.92)	30 (24.39)	
Previous history of HE, *n* (%)				0.494
No	378 (92.42)	266 (93.01)	112 (91.06)	
Yes	31 (7.58)	20 (6.99)	11 (8.94)	

HDL-C, high-density lipoprotein-cholesterol; LDL-C, low-density lipoprotein-cholesterol; APTT, activated partial thromboplastin time; PNI, prognostic nutritional index; MELD, model for end-stage liver disease; CTP, Child–Turcotte–Pugh; HE, hepatic encephalopathy.

### Screening of prognostic factors

In the training cohort, univariate Cox regression analysis indicated that age, hypertension, history of ascites, history of hepatic encephalopathy, total cholesterol, HDL-C, AST, serum sodium, BUN, creatinine, total bilirubin, albumin, serum ammonia, cholinesterase, and PT were significantly associated with prognosis. Variables with *p* < 0.05 were included in the multivariate Cox model. Ultimately, the multivariate analysis identified six independent prognostic factors: age (HR = 1.06, 95% CI: 1.04–1.09, *p* < 0.001), creatinine (HR = 1.02, 95% CI: 1.00–1.03, *p* = 0.009), TC (HR = 1.01, 95% CI: 1.00–1.02, *p* = 0.039), TBIL (HR = 1.01, 95% CI: 1.00–1.02, *p* = 0.001), albumin (HR = 0.93, 95% CI: 0.88–0.99, *p* = 0.021), and serum ammonia (HR = 1.01, 95% CI: 1.00–1.02, *p* = 0.003) (Table 2). The variance inflation factor for all six independent predictors was less than 5, indicating no significant multicollinearity in our final model (Supplementary Table S1).

**Table 2:** Univariable and multivariable cox regression analyses of factors affecting postoperative survival following TIPS.

Characteristic	Univariate analysis HR (95% CI)	*p*-value	Multivariate analysis HR (95% CI)	*p*-value
Age	1.07 (1.04–1.09)	<0.001	1.06 (1.04–1.09)	<0.001
Gender	0.88 (0.53–1.49)	0.642	—	—
BMI	1.00 (0.98–1.02)	0.881	—	—
Smoking	0.89 (0.51–1.55)	0.677	—	—
Drink	1.07 (0.64–1.78)	0.808	—	—
Diabetes	1.43 (0.79–2.6)	0.239	—	—
Hypertension	2.35 (1.37–4.05)	0.002	1.42 (0.72–2.74)	0.315
Previous history of gastrointestinal hemorrhage	0.62 (0.37–1.04)	0.069	—	—
Previous history of ascites	1.75 (1.05–2.92)	0.031	1.23 (0.69–2.19)	0.477
Previous history of HE	2.31 (1.04–5.09)	0.04	1.47 (0.5–4.36)	0.487
White blood cell count	1.05 (0.99–1.11)	0.08	—	—
Red blood cell	0.77 (0.58–1.03)	0.745	—	—
Hemoglobin	1.00 (0.99–1.01)	0.681	—	—
Platelet count	1.00 (0.98–1.00)	0.345	—	—
Neutrophil	1.04 (0.98–1.10)	0.154	—	—
Lymphocyte	0.86 (0.56–1.33)	0.503	—	—
Monocyte	1.06 (0.85–1.33)	0.959	—	—
Serum sodium	0.88 (0.83–0.94)	<0.001	0.93 (0.86–1.01)	0.077
Urea nitrogen	1.03 (1.01–1.06)	0.008	0.92 (0.83–1.00)	0.063
Creatinine	1.01 (1.00–1.01)	<0.001	1.02 (1.00–1.03)	0.009
Total cholesterol	1.01 (1.00–1.02)	0.006	1.01 (1.00–1.02)	0.039
Triglyceride	0.85 (0.51–1.42)	0.539	—	—
HDL-C	0.43 (0.19–0.97)	0.041	1.03 (0.42–2.52)	0.941
LDL-C	0.80 (0.53–1.21)	0.285	—	—
Alanine aminotransferase	1.00 (0.99–1.01)	0.886	—	—
Aspartate aminotransferase	1.00 (1.00–1.01)	0.034	1.00 (0.99–1.01)	0.774
Total bilirubin	1.01 (1.01–1.01)	<0.001	1.01 (1.00–1.02)	0.001
Total protein	0.98 (0.95–1.01)	0.106	—	—
Albumin	0.91 (0.88–0.94)	<0.001	0.93 (0.88–0.99)	0.021
Alkaline phosphatase	1.00 (1.00–1.01)	0.111	—	—
Gamma-glutamyl transferase	1.00 (1.00–1.01)	0.875	—	—
Total bile acid	1.01 (1.00–1.01)	<0.001	1.00 (0.99–1.01)	0.982
Cholinesterase	0.99 (0.99–1.00)	0.007	1.00 (0.99–1.00)	0.678
Serum ammonia	1.01 (1.01–1.02)	<0.001	1.01 (1.00–1.02)	0.003
Lactate dehydrogenase	1.00 (0.99–1.01)	0.029	1.00 (0.99–1.00)	0.250
Prothrombin time	1.09 (1.02–1.16)	0.008	0.99 (0.91–1.09)	0.856
APTT	1.00 (0.99–1.01)	0.875	—	—

### Construction of the nomogram model

**Figure 2:**
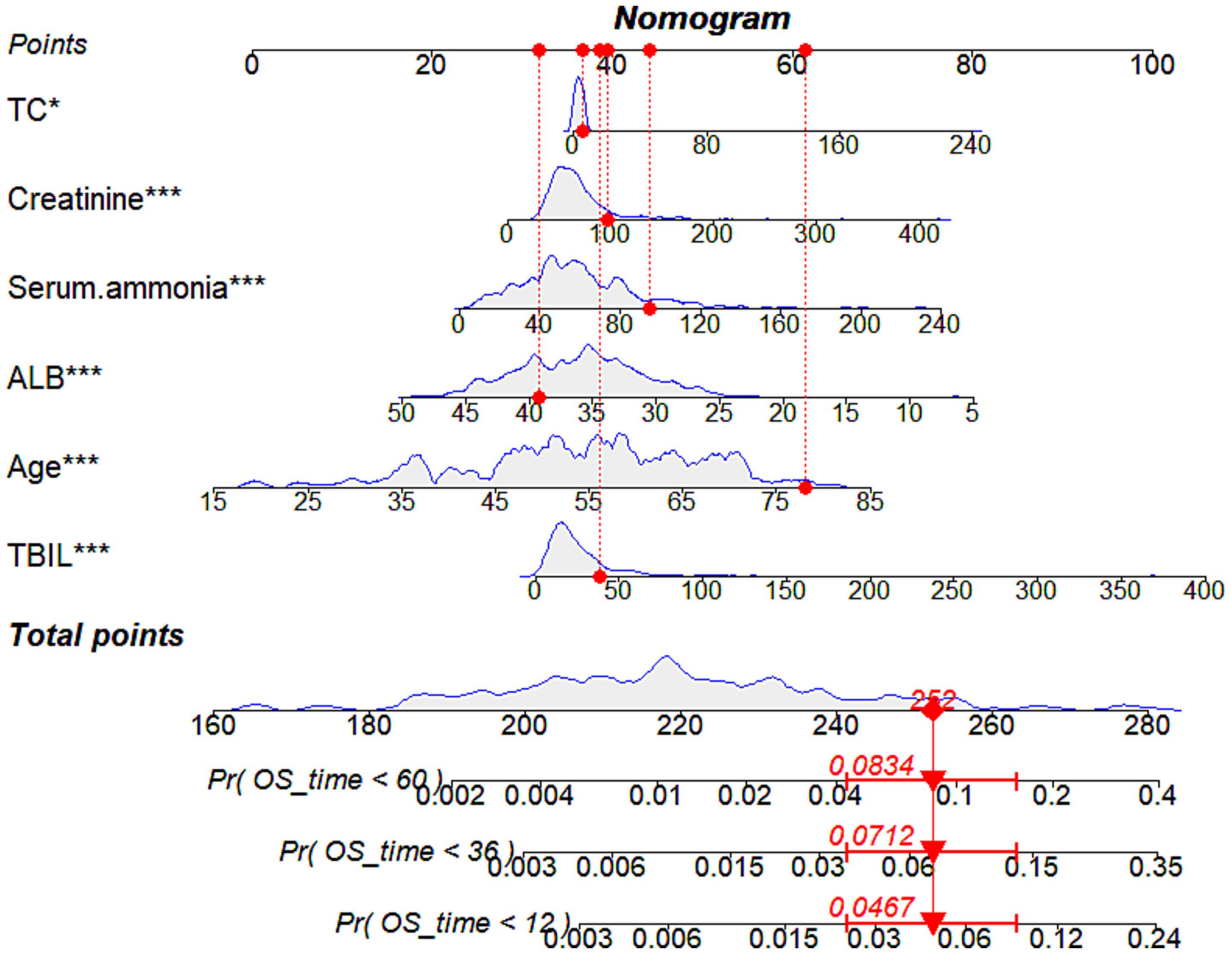
Nomogram for predicting 1-, 3-, and 5-year overall survival in patients with decompensated cirrhosis after TIPS. For clarity, the time unit for OS_time is months. The prediction axes at the bottom, denoted as Pr(OS_time <12), Pr(OS_time <36), and Pr(OS_time <60), represent the cumulative probability of death (i.e., mortality risk) occurring before 12, 36, and 60 months, respectively, rather than the survival probability. Therefore, it is mathematically and clinically expected that the cumulative mortality risk increases over time (e.g., the 60-month risk is higher than the 12-month risk).

To provide a visual and individualized prediction of post-TIPS survival, a nomogram was constructed based on the six independent risk factors identified (Figure 2). The model integrates age, TBIL, TC, Cr, albumin, and serum ammonia. Each indicator’s value range corresponds to a specific point score. Clinicians can locate the patient’s value on each axis, draw a vertical line to the “Points” axis to obtain the score, and sum these to get the “Total Points.” A vertical line drawn from the total points axis to the survival axes provides the predicted 1-, 3-, and 5-year survival probabilities.

### Performance validation and comparison

**Figure 3:**
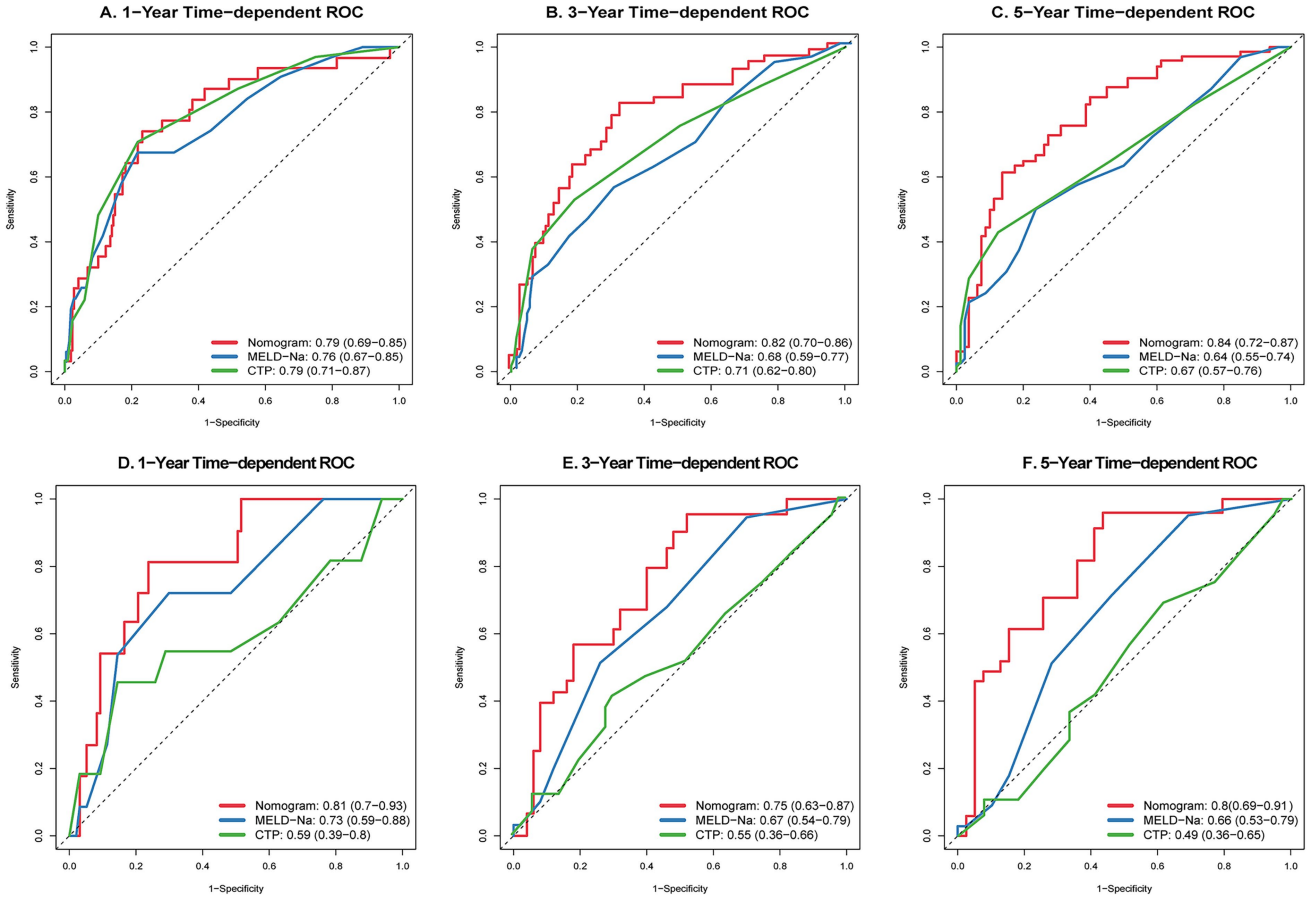
Comparison of time-dependent ROC curves between the novel nomogram, MELD-Na, and CTP scores. Training cohort: panels **(A–C)** present the 1-year, 3-year, and 5-year survival predictions, respectively. Validation cohort: panels **(D–F)** present the 1-year, 3-year, and 5-year survival predictions, respectively.

Time-dependent ROC curves were plotted to assess performance at specific time points. In the training cohort, the AUCs for 1-, 3-, and 5-year survival were 0.79, 0.82, and 0.84, respectively. In the validation cohort, the model demonstrated robust discrimination with AUCs of 0.81, 0.75, and 0.80, respectively. Notably, the nomogram’s AUCs consistently outperformed those of MELD-Na and CTP scores at corresponding time points in both cohorts (Figure 3).

### Calibration and clinical utility

**Figure 4:**
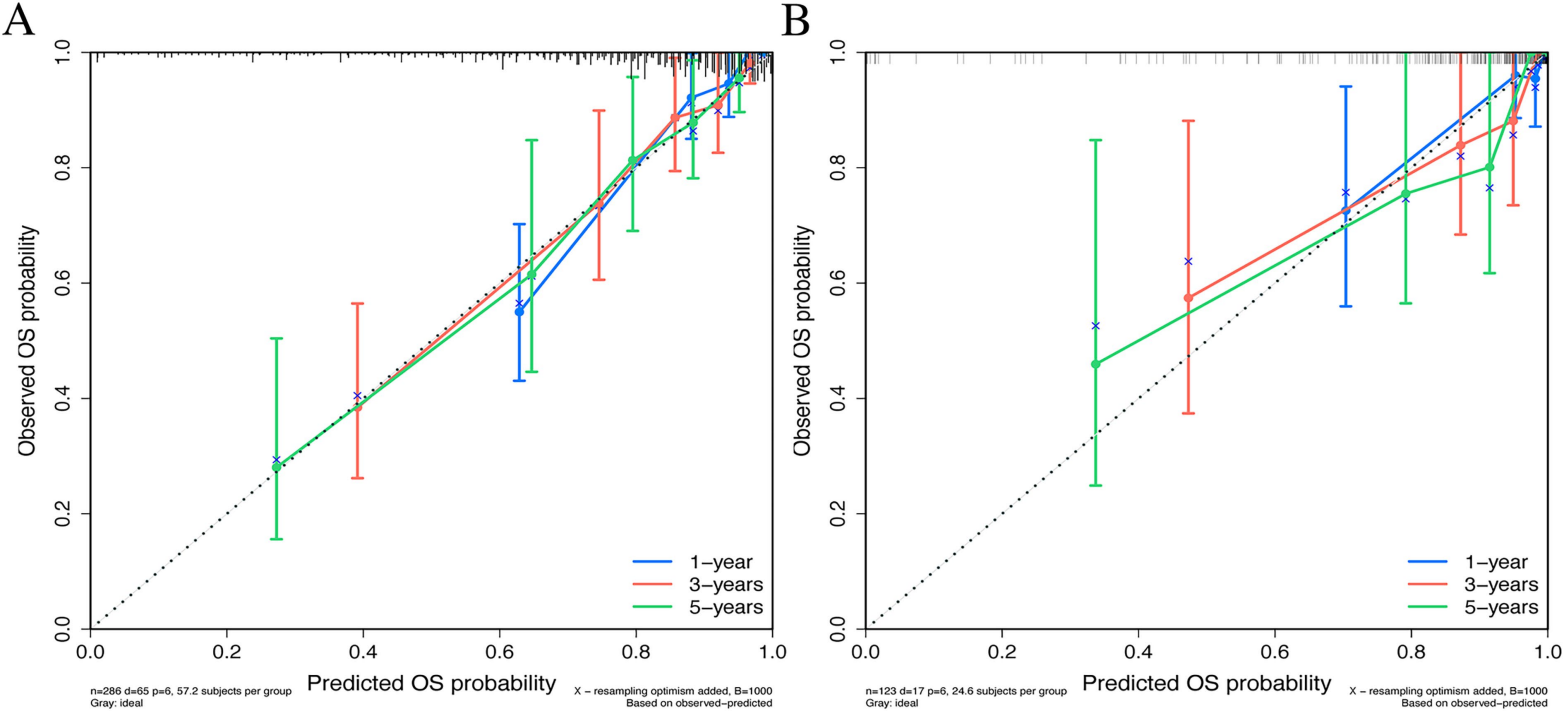
Calibration curves of the nomogram for 1-, 3-, and 5-year survival predictions. **(A)** Training cohort. **(B)** Validation cohort.

**Figure 5:**
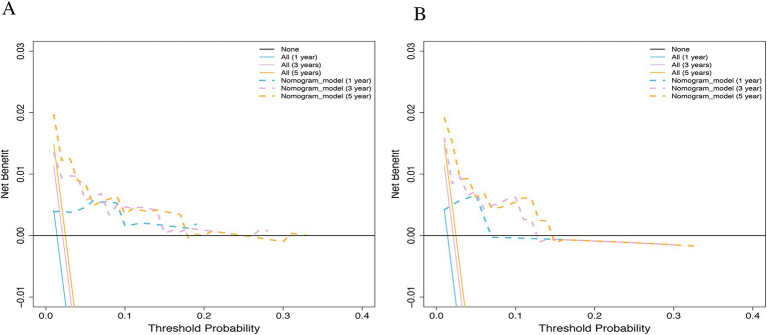
Decision curve analysis (DCA) for the 1-, 3-, and 5-year survival nomogram. **(A)** Training cohort. **(B)** Validation cohort.

Calibration curves showed high consistency between predicted and observed survival probabilities at 1, 3, and 5 years in both the training (Figure 4A) and validation (Figure 4B) cohorts, aligning closely with the ideal 45-degree diagonal. Decision curve analysis (DCA) demonstrated that the nomogram provided a higher net clinical benefit than “treat-all” or “treat-none” strategies across a wide range of threshold probabilities in both cohorts (Figures 5A,B), indicating practical value for clinical decision-making.

### Risk stratification

**Figure 6:**
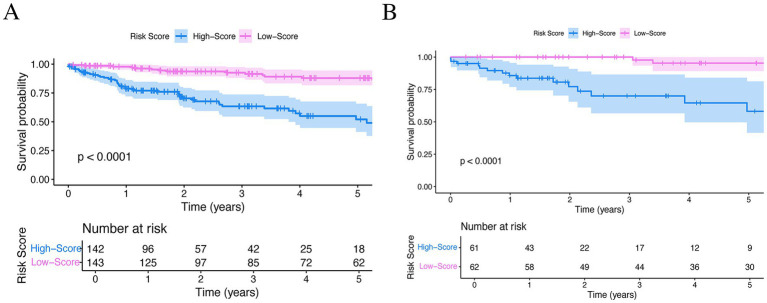
Kaplan–Meier survival curves based on risk stratification. **(A)** Training cohort. **(B)** Validation cohort.

Patients were stratified into high-risk and low-risk groups based on the median total nomogram score. Kaplan–Meier analysis revealed that the high-risk group had significantly lower overall survival compared to the low-risk group in the training cohort (*p* < 0.0001) (Figure 6A). This finding was successfully replicated in the validation cohort (*p* < 0.0001) (Figure 6B), confirming the model’s robust risk stratification capability.

## Discussion

This study, based on a single-center retrospective cohort, systematically evaluated factors influencing long-term survival after TIPS in patients with cirrhosis. We constructed a nomogram prediction model incorporating six indicators: age, total cholesterol, albumin, serum ammonia, creatinine, and total bilirubin. The results demonstrate that this model performs well in terms of discrimination, calibration, and clinical utility, consistently outperforming the clinical gold-standard MELD-Na and traditional CTP scores. This study offers a novel tool for pre-TIPS risk stratification and individualized decision-making, with high potential for clinical translation.

Recently, researchers have developed targeted risk prediction models for TIPS patients to address the shortcomings of traditional scoring systems. Bettinger et al. (10) proposed the Freiburg index of post-TIPS survival (FIPS) based on age, bilirubin, creatinine, and INR, which showed good performance for early mortality in the training set. However, due to a lack of long-term follow-up, FIPS is primarily used for predicting 90-day survival. Similarly, other post-TIPS models (13–16) have shown good efficacy for short-term prediction. In contrast, our nomogram provides survival predictions for 1, 3, and 5 years, offering greater clinical utility for long-term management and follow-up strategies. Furthermore, the FIPS model was derived from a German cohort predominantly consisting of alcoholic or non-alcoholic steatohepatitis patients. In China, the primary etiology for TIPS candidates is viral hepatitis. Significant differences in disease course, metabolic background, and complications between these populations suggest that directly applying FIPS may lead to prediction bias, highlighting the necessity of building models based on local population data.

Beyond differences in etiology and prediction horizons, our nomogram diverges fundamentally from FIPS in its core model construction concept. FIPS essentially represents an optimization of the traditional MELD paradigm; by relying heavily on acute excretory and synthetic failure markers (such as INR and bilirubin), it excels at identifying patients too critically ill to survive the immediate perioperative phase. In contrast, our model shifts the conceptual focus toward a “Systemic Reserve and Toxic Shunting” paradigm. By explicitly omitting INR and substituting it with albumin and total cholesterol, we capture the chronic nutritional depletion and metabolic paralysis that dictate long-term frailty. More crucially, our model incorporates serum ammonia, which directly quantifies the specific pathophysiological consequence of the TIPS procedure itself—the portosystemic shunt. Therefore, while FIPS evaluates acute end-organ failure, our nomogram is conceptually engineered to assess a patient’s holistic biological capital to endure long-term post-TIPS hemodynamic and metabolic stress.

The innovation of this study lies in the integration of six easily accessible pre-operative clinical indicators—age, serum ammonia, total cholesterol, total bilirubin, albumin, and creatinine. These six factors synergistically depict a comprehensive pathophysiological landscape of patients with decompensated cirrhosis undergoing the TIPS procedure. Our findings are highly consistent with previous literature (3, 17, 18), suggesting that these six prognostic factors have a solid pathophysiological basis, and their combination comprehensively reflects the systemic imbalance in decompensated cirrhosis. We conceptualize the prognostic value of our nomogram through three interconnected pathophysiological axes.

First, the hepato-renal hemodynamic crosstalk axis (albumin and Cr). TIPS creation fundamentally alters systemic hemodynamics by directly shunting portal blood into the systemic circulation. The ability of a patient to tolerate this cardiovascular shock heavily depends on their intravascular volume maintenance and downstream organ perfusion. Albumin is critical for maintaining plasma colloid osmotic pressure and stabilizing microcirculation (19–21). Severe pre-operative hypoalbuminemia compromises effective arterial blood volume. When compounded by the sudden splanchnic vasodilation and post-TIPS hemodynamic shifts, it severely predisposes patients to circulatory dysfunction. This hemodynamic instability directly strikes the kidneys, the most perfusion-sensitive end-organ. Elevated creatinine (Cr) is the hallmark of this disrupted hepato-renal crosstalk, often culminating in hepatorenal syndrome-acute kidney injury (22, 23). Therefore, the combination of albumin and Cr in our model intimately reflects the systemic hemodynamic fragility and the imminent risk of multi-organ hypoperfusion post-TIPS.

Second, the metabolic paralysis and toxic shunting axis (TBIL, TC, and serum ammonia). Decompensated cirrhosis is characterized by an overwhelming metabolic and toxic burden. Elevated total bilirubin (TBIL) and aberrant total cholesterol (TC) levels are direct indicators of profound hepatocellular synthetic and excretory failure, as well as the activation of systemic metabolic inflammation (24–26). Failing hepatocytes struggle to manage lipid metabolism and bile excretion, creating a highly pro-inflammatory state. Crucially, the TIPS stent acts as a physical bypass, allowing gut-derived toxins—most notably serum ammonia—to evade the remaining, already compromised hepatic urea cycle (27, 28). The confluence of severe intrinsic hepatocellular paralysis (high TBIL/TC) and massive exogenous toxic shunting (high serum ammonia level) unleashes systemic neurotoxicity and neuroinflammation, precipitating severe hepatic encephalopathy (HE) and sharply increasing the risk of mortality.

Finally, age acts as the overarching systemic frailty multiplier. Age is not merely a chronological metric but a reflection of the patient’s ultimate physiological reserve (3, 18). Advanced age correlates with “immunosenescence” and “inflammaging,” leading to a chronic, low-grade inflammatory state that severely impairs liver regeneration. Senescent hepatocytes and hepatic stellate cells exhibit aberrant repair responses, favoring fibrosis over effective regeneration (29). Consequently, an older patient has significantly diminished physiological capital to buffer the hepato-renal shock (albumin/Cr axis) and the metabolic-toxic onslaught (TBIL/TC/serum ammonia axis) induced by TIPS.

By integrating these axes, our nomogram transcends a simple mathematical combination of variables; it serves as a holistic mirror reflecting the intricate systemic imbalance, hemodynamic vulnerability, and multi-organ functional exhaustion in end-stage liver disease.

Beyond discriminative accuracy, a clinically valuable model must demonstrate practical benefit in decision-making. While our DCA confirmed the clinical utility of the nomogram, two aspects warrant detailed discussion: the specific threshold probability range and the magnitude of the absolute net benefit. First, the model exhibited positive net benefit primarily within a threshold probability range of 0.1 to 0.2. In the context of fatal outcomes like post-TIPS mortality, clinicians generally adopt a low threshold for intervention. A 10 to 20% predicted risk of death represents the critical “grey zone” where early interventions are actively considered. Thus, the model provides decision support precisely where clinical uncertainty is highest. Second, the absolute net benefit was relatively small (approximately 0.01). However, it is mathematically constrained by the overall baseline mortality rate of the cohort. Moreover, in time-dependent survival analysis, the inherent presence of right-censored patients further suppresses the cumulative observed event rate at specific time points, inherently capping the absolute net benefit value. Clinically, an absolute net benefit of 0.01 translates to correctly identifying one additional high-risk patient per 100 evaluations without increasing the rate of false-positive interventions. Given the grim prognosis of end-stage liver disease, this incremental improvement in risk stratification facilitates early, targeted interventions, providing tangible clinical significance despite the numerically modest absolute net benefit.

Beyond its discriminative accuracy and statistical net benefit, the ultimate value of this nomogram lies in its capacity to genuinely alter clinical decision-making and optimize resource allocation. By calculating a patient’s total score, clinicians can seamlessly stratify candidates into low- or high-risk categories based on the established cutoff threshold (the median score of the cohort). This risk stratification provides actionable clinical guidance in two critical domains. First, in pre-operative decision-making, a high-risk score serves as a vital prognostic warning. Particularly for patients with elective TIPS indications such as refractory ascites, a high score suggests that the substantial risk of post-operative liver failure and severe hepatic encephalopathy may outweigh the intended benefits of portal decompression. In such scenarios, the nomogram alerts the multidisciplinary team to potentially avoid or delay TIPS placement, prompting an expedited evaluation for liver transplantation or a shift toward conservative palliative care. Second, regarding post-operative management, the nomogram proactively guides individualized follow-up strategies. Patients categorized into the high-risk group necessitate an intensive surveillance protocol. This includes highly frequent Doppler ultrasounds to monitor shunt patency, rigorous neurological assessments for early signs of encephalopathy, and aggressive prophylactic medical management (e.g., lactulose and rifaximin). Conversely, low-risk patients can safely adhere to standard follow-up intervals.

### Limitations

This study has limitations. First, the single-center retrospective design may introduce selection bias. Second, although internal validation demonstrated good performance, the sample size is relatively limited. The model’s generalizability requires further verification in larger-scale, multi-center prospective cohorts to ensure stable predictive value across different populations and clinical settings.

## Conclusion

The post-TIPS prognostic model established in this study integrates multi-dimensional pre-operative indicators and outperforms traditional scoring systems in predictive capability and interpretability. It comprehensively reflects the patient’s overall compensatory state and multi-organ functional reserve. This model serves as a reliable tool for pre-operative individualized survival assessment and risk stratification, demonstrating excellent operability and potential for clinical application.

## Data Availability

The original contributions presented in the study are included in the article/Supplementary material, further inquiries can be directed to the corresponding authors.
