# Supplementary material for: Development and validation of a novel nomogram for predicting long-term survival in patients with decompensated cirrhosis undergoing TIPS
Source: Front Med (Lausanne). 2026 Apr 7;13:1766352. doi: 10.3389/fmed.2026.1766352 (PMC13096019; doi:10.3389/fmed.2026.1766352)
Supplement: Supplementary file 1 [file Table_1.docx]

**Supplementary Table S1. Variance Inflation Factor (VIF) of the predictors in the final model**.

| Variables | VIF |
| --- | --- |
| Age | 1.12 |
| TC | 1.05 |
| TBIL | 1.34 |
| Serum ammonia | 1.21 |
| Creatinine | 1.15 |
| ALB | 1.08 |
